# An Accord of Nuclear Receptor Expression in *M. tuberculosis* Infected Macrophages and Dendritic Cells

**DOI:** 10.1038/s41598-018-20769-4

**Published:** 2018-02-02

**Authors:** Ankita Saini, Sahil Mahajan, Nancy Ahuja, Ella Bhagyaraj, Rashi Kalra, Ashok Kumar Janmeja, Pawan Gupta

**Affiliations:** 1 Institute of Microbial Technology, Council of Scientific and Industrial Research, Chandigarh, 160036 India; 2 Government Medical College and Hospital, Chandigarh, 160030 India; 3 Present Address: Department of Pathology and Immunology, Washington University School of Medicine, St. Louis, MO 63110 USA; 4 Present Address: Department of Orthopedics, Washington University School of Medicine, St. Louis, MO 63110 USA

## Abstract

*Mycobacterium tuberculosis* instigates interactions with host factors to promote its survival within the host inimical conditions. Among such factors, nuclear receptors (NRs) seem to be promising candidates owing to their role in bacterial pathogenesis. However, only few members of NR superfamily have been implicated in *M. tuberculosis* infection and there is a dearth of comprehensive knowledge about expression or function of the entire superfamily. In this study, we performed detailed expression analysis and identified key NRs getting differentially regulated in murine macrophages and dendritic cells (DC) upon infection with *H37Rv*. The murine macrophages and DCs infected with *H37Rv* entailed overlapping changes in the expression of certain NRs which reflect upon the possibility that both cells might utilize similar transcriptional programs upon *M. tuberculosis* infection. We identified Nr4a3 and Rora, which have not been implicated in *M. tuberculosis* pathogenesis, undergo similar changes in expression in macrophages and DCs upon *H37Rv* infection. Interestingly, a similar pattern in their expression was also observed in infected human monocyte derived macrophages and the findings corroborated well with PBMCs obtained from TB patients. This all-inclusive analysis provides the basis for a precise approach in identifying NRs that can be targeted therapeutically in intracellular bacterial infections.

## Introduction

The cells of innate immune system particularly macrophages and dendritic cells (DCs) provide the first line of defence against *Mycobacterium tuberculosis* and they are among the first few cells to interact with the bacteria upon infection in the host. Macrophages and DCs utilize a wide variety of surface receptors including toll-like receptors, opsonic receptors, mannose receptors, etc. that recognize antigenic molecules on the bacterial surfaces^1–5^. Engagement of bacterial pathogen-associated molecular patterns (PAMPs) with these host cell receptors results in the bacterial uptake and entrapment of bacteria in the phagosomes. The members of nuclear receptor (NR) superfamily not only regulate the signalling through such pathogen recognition receptors but have also been shown to sense the lipid components of mycobacteria by their direct binding to such lipids^6,7^. The NR superfamily that comprises a total of 49 members has been categorized into three families namely, endocrine, adopted orphan and orphan NRs on the basis of the nature of the ligand they bind^8–11^.

A growing literature about NRs in infectious diseases highlights that they are important in shaping up the innate immune responses and several pathogens, for their own benefits, have evolved strategies to modulate NRs either by changing their expression or by interfering with their transcriptional activity. For example, *Listeria monocytogenes* and *M. tuberculosis* induce the expression of peroxisome proliferator-activated receptor gamma (Pparg) in macrophages^12–14^. Expression of Pparg is also elevated in response to a PAMP called mannose-capped lipoarabinomannan obtained from the cell wall of *M. tuberculosis*^15^. Interestingly, silencing of Pparg in macrophages significantly reduces the bacterial burden supporting the pro-bacterial role for Pparg^12^. In addition to Pparg, another NR named testicular receptor 4 (Tr4) also acts as a molecular switch and by regulating the macrophage polarization towards M2 phenotype, augments the bacterial survival^12,16^. The infection with intracellular bacteria like *M. tuberculosis*, *L. monocytogenes* and *Shigella flexneri* also elevates the levels of liver X receptor alpha (Lxra) in macrophages^17,18^. Moreover, infected macrophages have elevated levels of Lxra target genes suggesting that transcriptional programs induced by Lxra promote the clearance of bacteria inside the macrophages^17^. In agreement with these findings, Lxra deficient mice showed increased susceptibility to bacterial infections^17,18^. Also, along with Lxr the binding sites of retinoic acid receptor (Rar) are enriched in H3K4me1 regions (a marker for active or poised enhancers) in *M. tuberculosis* infected THP-1 cells^19^. In accordance with these findings, studies performed on human DCs infected with *M*. *tuberculosis* also revealed enrichment of Lxr and Rar binding sites^20^. Furthermore, the transcriptomic analysis of blood samples from active pulmonary TB patients revealed an altered expression in vitamin D receptor (Vdr) associated genes compared to the individuals with latent bacterial infection. Vdr-regulated genes are also enriched in *M. tuberculosis* infected THP-1 cell line^21,22^. Many other members of NR superfamily which includes glucocorticoid receptor (Gr), pregnane X receptor (Pxr), Nr1d1, and Vdr have also been shown to be involved in the pathogenesis of bacterial infection^23–28^. Furthermore, NRs are amicable to modulation by small molecules (which are either available or can be synthesized easily) and hence numerous endogenous and synthetic NR ligands have been evaluated in various infection models as potential therapies^29^.

*M*. *tuberculosis* is unique in its ability as it successfully parasitizes both macrophages and DCs, which in traditional view are considered as host sentinels for initiating protective immunity against bacterial infections. With the emergence of knowledge about the cross-talk between host NRs and *M*. *tuberculosis*, it is compelling to identify all those NRs which have a bearing on the persistence and pathogenesis of mycobacteria in macrophages and DCs. A composite gene expression analysis of NRs performed in macrophages and DCs has suggested a regulatory role of NR superfamily in macrophages and DC functionality^8,30^. However, these studies have examined the expression of these NRs only in the presence of a PAMP or an immunological modulator and we still need to unravel a composite list of all NRs being modulated by mycobacteria in its innate immune cell niches. In this study, we performed a detailed expression profiling of NR superfamily in murine macrophages and DCs upon *M. tuberculosis* infection. In conjunction with identifying NRs whose role in *M. tuberculosis* pathogenesis has not yet been established we also recapitulated some of the findings which have been previously published. Intriguingly, we observed that NRs such as Vdr, Lxra, Pparg, Rarg, Nr4a’s, RAR-related orphan receptor alpha (Rora), androgen receptor (Ar), and thyroid hormone receptor beta (Thrb) were undergoing differential regulation in both macrophages and DCs upon *M. tuberculosis* infection suggesting a plausible cross-talk between these NRs and *M. tuberculosis*. Interestingly, our analysis also revealed Rora and Nr4a3, both of which have not been previously implicated in TB infection, undergo changes in expression upon *M. tuberculosis* infection. We identified that *H37Rv* induces the expression of Rora while the expression of Nr4a3 was significantly reduced in murine and human macrophages. We also observed a similar pattern in the expression of Rora and Nr4a3 in murine DCs and PBMCs obtained from patients with active TB. Therefore, examining the potential of ligands for such NRs as an approach to anti-TB therapy could be promising.

## Results

### Identification of NRs modulated by *M. tuberculosis H37RV* in mBMDCs

**Figure 1:**
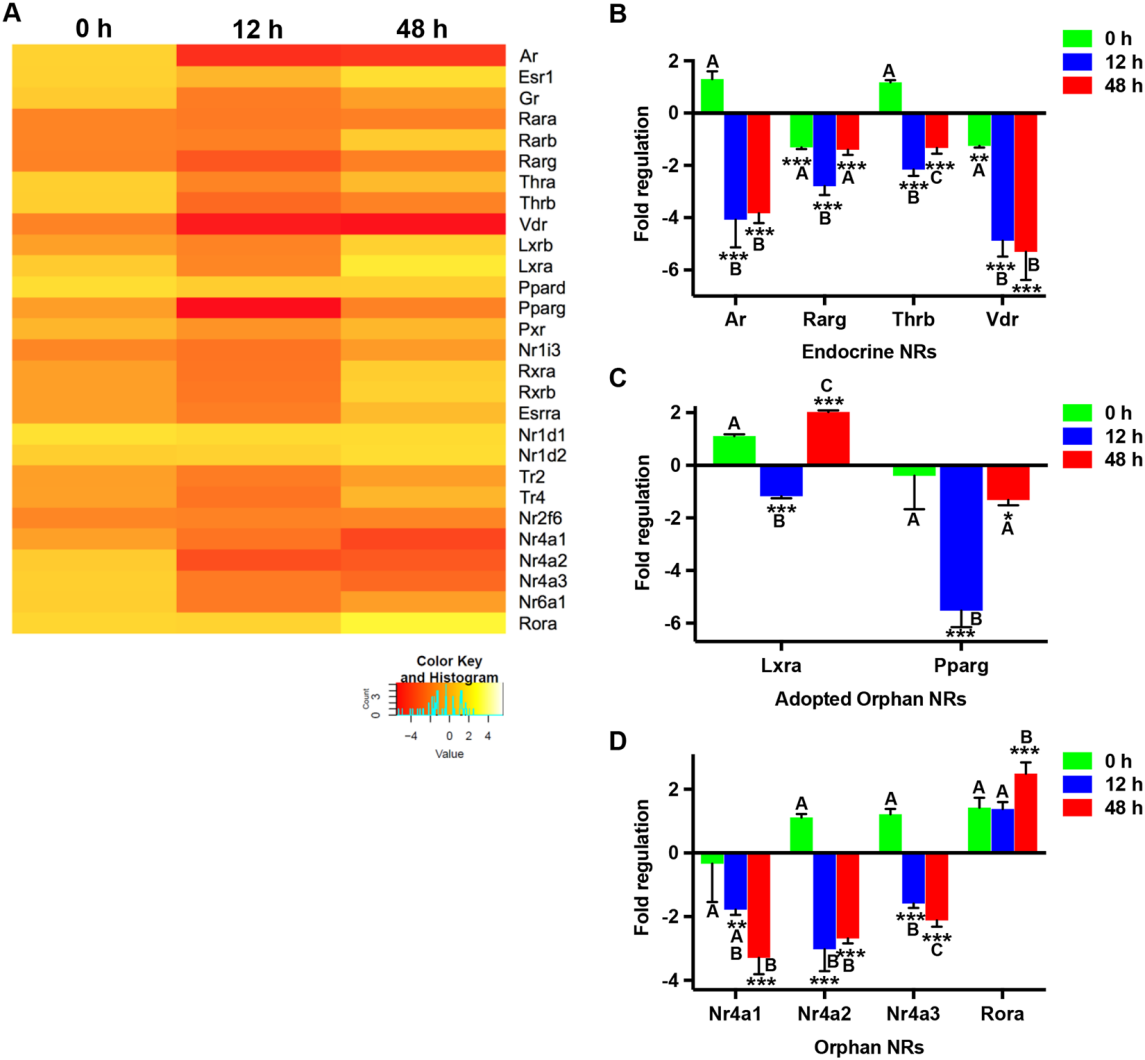
Mapping NRs expression profile in mBMDCs infected with mycobacterium *H37Rv*. (**A**) Heatmap depicting the fold expression of NRs in mBMDCs at 0 h, 12 h and 48 h after *H37Rv* infection, relative to uninfected mBMDCs. Transcripts with Ct values 32 or less are presented. Yellow and red colour displays the up- and down-regulated genes respectively as illustrated in the scale bar below the heatmap. Average fold regulation showing atleast two-fold change for (**B**) endocrine, (**C**) adopted orphan and (**D**) orphan NRs at different time points post *H37Rv* infection relative to uninfected mBMDCs are plotted. Results are mean and s.d. of three independent experiments. *p < 0.05, **p < 0.01 and ***p < 0.001, versus uninfected mBMDCs (one-way ANOVA). Letters above bars depict connecting letter report representing correlation of NRs expression at different time points. Bars not connected by the same letter are significantly different.

Being an intracellular pathogen, *M. tuberculosis* resides in cell types such as macrophages and DCs. Among these, macrophages have been largely the focus for studying cross-talk of *M. tuberculosis* with the host. However, given the unique ability of DCs to initiate T cell responses, DCs could possibly regulate the adaptive immune responses against the bacteria. Recently DCs have been shown to express certain repertoire of NRs, few of which have also been shown to regulate DC functions^8,31,32^. So, in order to address whether this pathogen has the ability to modulate any of these NRs in DCs and thereby DC function, in this study, we infected mouse bone marrow-derived DCs (mBMDCs) with *H37Rv* and monitored the expression of NRs relative to uninfected control cells. We selected 0 h, 12 h, and 48 h post infection as the time points which allowed us to identify the changes in the transcript levels of NRs during early and later stages of infection. Interestingly, we found that the levels of some NRs were being altered upon infection (Fig. 1A). Among the endocrine NRs, *Ar*, *Rarg*, *Thrb*, and *Vdr* underwent significant downregulation of greater than 2-folds (Fig. 1B). While *Ar* and *Vdr* remained constantly repressed during later time points of infection, the expression of *Rarg* and *Thrb* started to rescue at 48 h post infection. The adopted orphan NRs, *Lxra* and *Pparg* were downregulated at 12 h (Fig. 1C) followed by an enhanced expression of *Lxra* and recovery of *Pparg’s* basal expression at 48 h after infection. In the orphan NR family, all three members of Nurr family (*Nr4a1*, *Nr4a2* and *Nr4a3*) exhibited repression while *Rora* was upregulated during later stages of infection (Fig. 1D). Taken together, our data identifies the members of NR superfamily which are being significantly modulated in mBMDCs upon *M. tuberculosis* infection. Of these, Vdr, Lxra and Pparg have already been shown to have an explicit role in *M. tuberculosis* pathogenesis thereby indicating plausible implication of other altered NRs in *M. tuberculosis* infection.

### Expression analysis of NRs in mBMDMs upon *M*. *tuberculosis* infection

**Figure 2:**
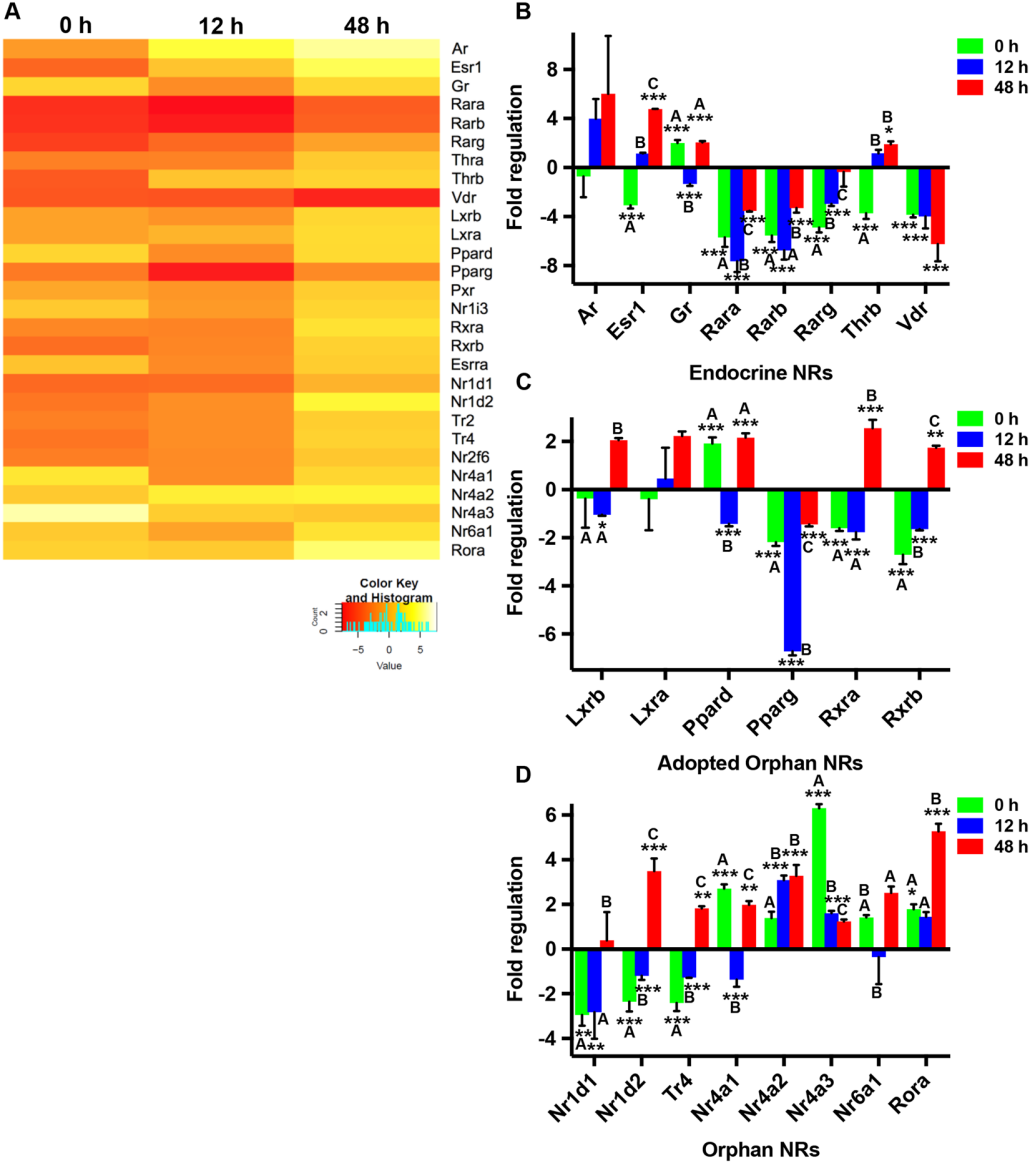
Mapping NRs expression profile in mBMDMs infected with mycobacterium *H37Rv*. (**A**) Heatmap depicting the fold expression of NRs in mBMDMs at 0 h, 12 h and 48 h after *H37Rv* infection, relative to uninfected mBMDMs. Transcripts with Ct values 32 or less are presented. Yellow and red colour displays the up- and down-regulated genes respectively as illustrated in the scale bar below the heatmap. Average fold regulation showing atleast two-fold change for (**B**) endocrine, (**C**) adopted orphan and (**D**) orphan NRs at different time points post *H37Rv* infection relative to uninfected mBMDMs are plotted. Results are mean and s.d. of three independent experiments. *p < 0.05, **p < 0.01 and ***p < 0.001, versus uninfected mBMDMs (one-way ANOVA). Letters above bars depict connecting letter report representing correlation of NRs expression at different time points. Bars not connected by the same letter are significantly different.

Given the modulation of NRs with an unidentified role in *M*. *tuberculosis* infection, in *H37Rv* infected mBMDCs, we next set out to profile entire NR superfamily in *H37Rv* infected mouse bone marrow-derived macrophages (mBMDMs) to further explore the members with a possible role in *M. tuberculosis* pathogenesis. A similar kind of analysis revealed that unlike mBMDCs, in mBMDMs certain NRs started responding during the early time point of infection (0 h). These included *Esr1*, *Gr*, *Rar’s*, *Thrb*, *Vdr*, *Pparg*, *Rxrb*, *Nr1d’s*, *Tr4*, *Nr4a1*, and *Nr4a3* (Fig. 2). At 48 h post infection, when the equilibrium between bacteria and macrophages was established, few NRs that did not respond during early infection (0 h) showed elevated levels (*Ppard*, *Rxra*, *Nr4a2*, and *Rora*). Furthermore, this analysis reflected that while some of the early responding NRs such as *Esr1* and *Nr1d2* had a reverse pattern of their expression at 48 h, others simply recovered their basal levels (*Nr1d1*, *Tr4*, *Nr4a1*, and *Nr4a3*). Thus, *M. tuberculosis* infection affects the levels of different NRs in its innate immune reservoirs, but whether these induced changes in an individual receptor favours pathogen or host needs to be established.

### NRs commonly modulated in mBMDCs and mBMDMs by *M. tuberculosis*

**Figure 3:**
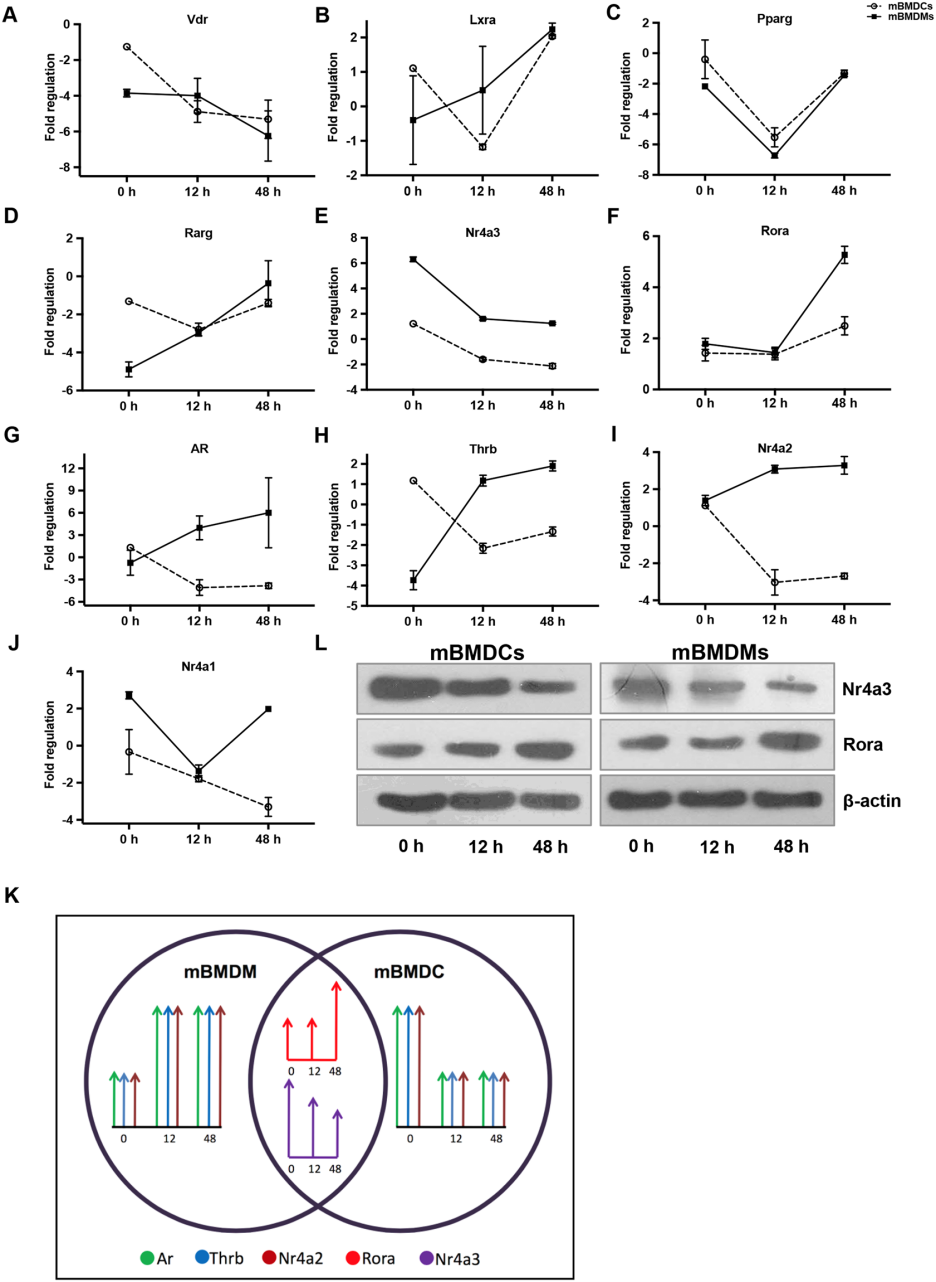
*M*. *tuberculosis* modulates NRs expression in murine macrophages and dendritic cells. NRs undergoing change in expression in both mBMDMs and mBMDCs are plotted. Comparative expression analysis of (**A**–**J**) NRs in mBMDMs and mBMDCs at 0 h, 12 h and 48 h post *H37Rv* infection. (**K**) A schematic representation showing NRs with significantly similar and opposite expression pattern in mBMDMs and mBMDCs at 0 h, 12 h and 48 h after *H37Rv* infection. (**L**) Immunoblot analysis of Nr4a3 and Rora in *H37Rv* infected mBMDCs and mBMDMs at 0 h, 12 h and 48 h post *H37Rv* infection. β-actin was used as loading control. The full length or source images of all immunoblots have been provided in the supplementary information. Results are mean and s.d. (**A**–**J**) or representative (**L**) of three independent experiments. *p < 0.05, **p < 0.01 and ***p < 0.001, versus uninfected mBMDMs or mBMDCs (one-way ANOVA).

Next, to additionally identify the key members of NR superfamily which might be crucial for *M*. *tuberculosis* pathogenesis we followed a systematic approach. Intriguingly, further in depth analysis revealed that *M. tuberculosis* was altering certain repertoire of NRs which was shared by both mBMDCs and mBMDMs. We identified that among the 49 members of the NR superfamily there were 10 NRs which were being modulated in both cell types upon *M. tuberculosis* infection (Fig. 3). Interestingly, six of them showed a similar pattern of regulation of their expression in mBMDCs and mBMDMs (Fig. 3A–F). Of these, *Vdr*, *Lxra* and *Pparg* have already been reported to have an implication in the tuberculosis disease. Whereas the role of others, which includes *Rarg*, *Nr4a3* and *Rora*, has not yet been identified and remains to be established in *M. tuberculosis* infection. On the contrary, *Ar* (Fig. 3G), *Thrb* (Fig. 3H), *Nr4a2* (Fig. 3I) and *Nr4a1* (Fig. 3J) exhibited upregulation in mBMDMs whereas their expression was downregulated in mBMDCs. The probable explanation for this could be the cell specific responses against mycobacteria in the studied cell types. As shown in Fig. 3K, *Rora* and *Nr4a3* have similar while *Ar*, *Thrb* and *Nr4a2* have different expression pattern in mBMDCs and mBMDMs. In corroboration, we observed similar modulation of *Rora* and *Nr4a3* at protein levels as well (Fig. 3L). Taken together, we speculated that these commonly modulated NRs could prove to be novel target molecules for developing drugs to control *M. tuberculosis* infection.

### Expression of NRs in CD11c positive cells isolated from lungs of *M. tuberculosis* infected mice

**Figure 4:**
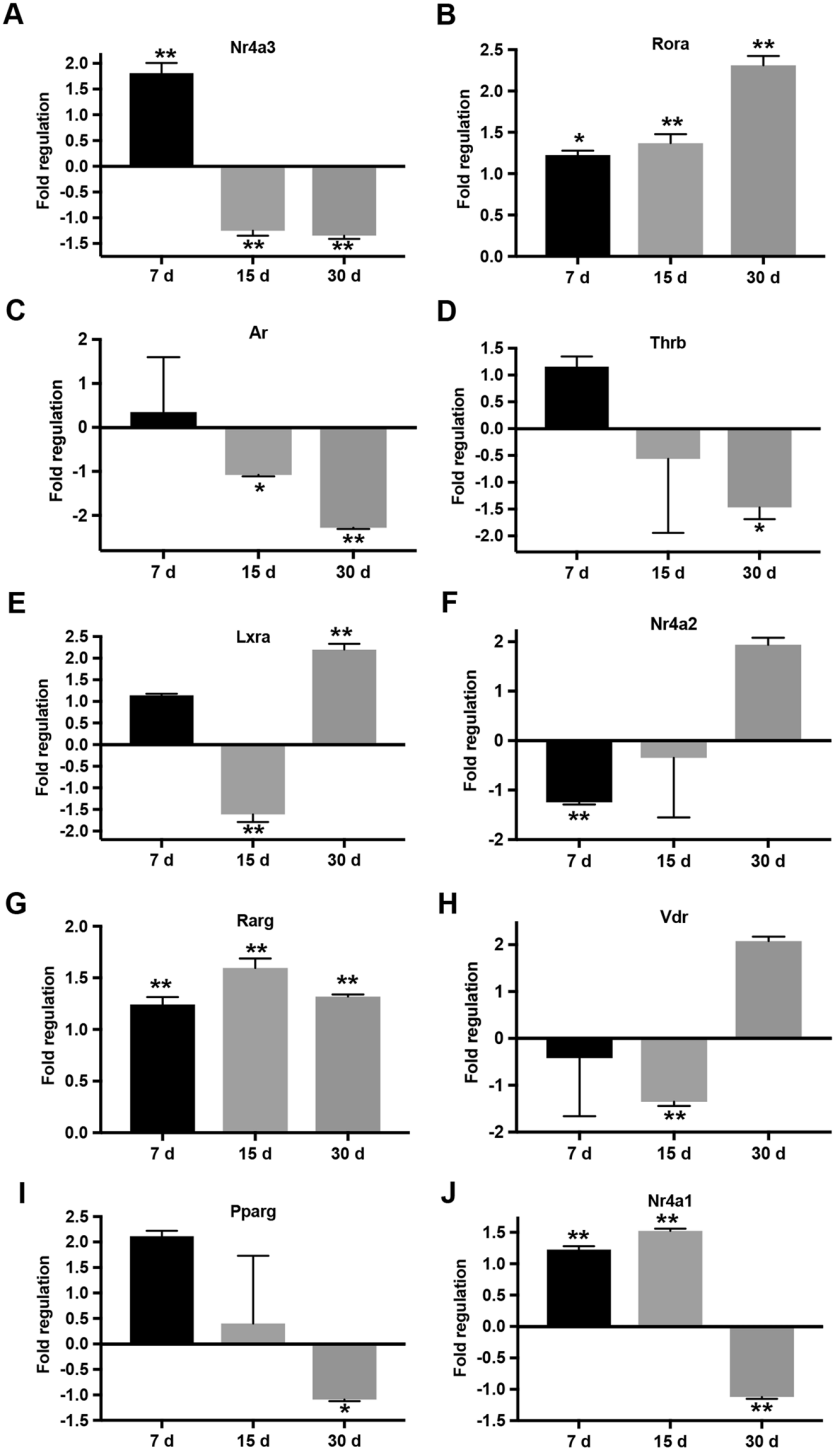
*M*. *tuberculosis* modulates NRs expression in CD11c positive cells. RT-qPCR analysis for relative expression of (**A**–**J**) NRs in CD11c positive cells isolated from lungs of *M. tuberculosis H37Rv* infected mice at day 7, 15, and 30 as compared to uninfected control mice. Target gene expression was normalized to β-actin and is presented as fold regulation relative to control. Results are mean and s.d. of three independent experiments.*p < 0.05 and **p < 0.01, versus control mice (one-way ANOVA).

To determine whether above identified commonly regulated NRs were also significant in an *in vivo* setting, we tested their levels in lungs isolated from the most commonly used mice model of *M. tuberculosis* infection. We selected day 7, 15 and 30 post aerosol infection for our analysis in order to study their kinetics over the early and later stages of infection. CD11c positive cells were isolated from the lungs of these mice which represented a cellular population consisting of alveolar macrophages and DCs. Interestingly, alteration in the expression of *Nr4a3* (Fig. 4A) and *Rora* (Fig. 4B) was in concordance with our *in vitro* findings. In addition, *Ar* (Fig. 4C), *Thrb* (Fig. 4D) and *Lxra* (Fig. 4E) reflected an expression pattern which was similar to mBMDCs, while *Nr4a2* (Fig. 4F) exhibited that of mBMDMs.

### Expression of Nr4a3 and Rora in hMDMs and clinical samples

**Figure 5:**
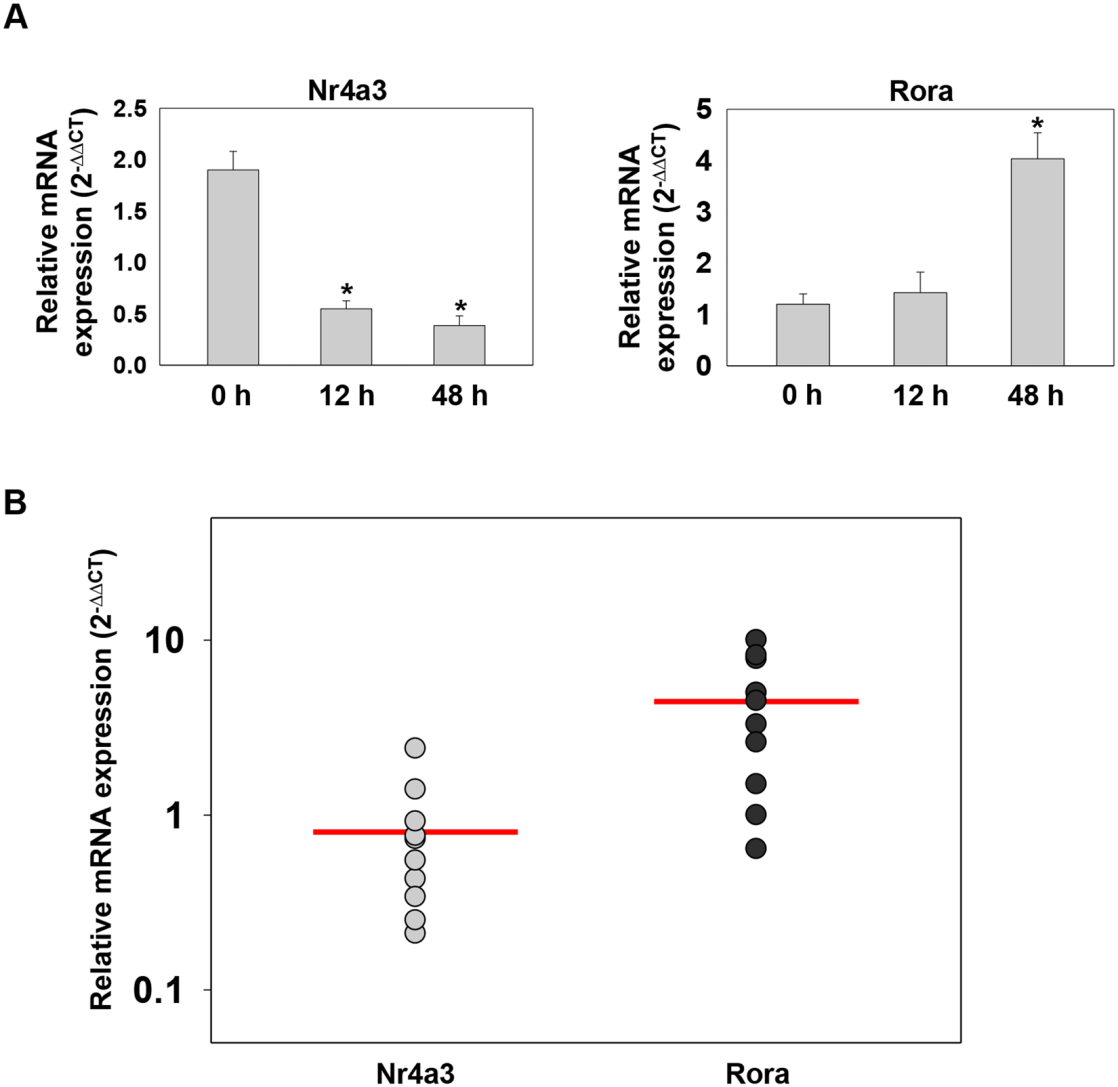
Expression analysis of Nr4a3 and Rora in hMDMs and clinical samples. (**A**) RT-qPCR analysis for relative expression of *Nr4a3* and *Rora* in *H37Rv* infected hMDMs isolated from healthy individuals at 0 h, 12 h and 48 h post infection. Results are mean and s.d. of three independent experiments. *p < 0.05, versus uninfected (one-way ANOVA). (**B**) Relative mRNA abundance of *Nr4a3* and *Rora* in human PBMCs isolated from freshly diagnosed TB patients (n = 10) was calculated as 2^−(ΔΔCt)^ relative to healthy individuals (n = 10).

To further extend our *in vitro* findings we monitored the expression of Nr4a3 and Rora in human monocyte derived macrophages (hMDMs) (obtained from healthy individuals). The hMDMs were infected with *H37Rv* and the levels of *Nr4a3* and *Rora* were measured at different time points (0, 12, and 48 h) (Fig. 5A). Consistent with our *in vitro* findings, we observed decreased expression of *Nr4a3* at 12 and 48 h post infection and increased expression of *Rora* at 48 h post infection. In addition, we also monitored relative mRNA abundance of *Nr4a3* and *Rora* in human PBMCs isolated from freshly diagnosed TB patients (Fig. 5B). We observed a relatively lesser expression of *Nr4a3* and higher expression of *Rora* in PBMCs isolated from TB patients compared to PBMCs isolated from healthy individuals. Altogether, these findings suggest that Nr4a3 and Rora might have an important role in *M. tuberculosis* pathogenesis.

## Discussion

*M*. *tuberculosis* gets transmitted via aerosol thereby lodging the lungs where it inhabits cells of different phenotypes as macrophages, DCs, neutrophils, monocytes, endothelial and epithelial cells. Among these, the innate immune cells are the principal cell type harbouring the bacteria inside the host body. The scientific challenges in understanding the immunology of *M. tuberculosis* arise from the observation that an apparently appropriate immune response is developed upon infection however, it generally leads to the establishment of a latent infection rather than elimination of pathogen^33,34^. In addition, *M. tuberculosis* outsmarts host defence mechanisms, manipulates host signalling mechanisms and overturns them for its own survival within the host^35^. The pathogen has a unique repertoire of cell wall lipids which have been shown to possess immunomodulatory properties and are important for its pathogenesis^36^. Earlier studies have shown that these lipids help the bacteria to invade the host cells and in subverting immune responses by modulating phagosome maturation, cytokine response, and antigen presentation^37^. In addition, *M. tuberculosis* actively sheds its lipids in the host cell which then enter the endocytic network and are trafficked within as well as outside the infected cell^38–41^. It has been proposed that microvesicles released in the extracellular environment by the infected host cells are taken up by the uninfected bystander cells including macrophages and DCs^39,40^. Among these, DCs in particular can migrate from the lungs to the local draining lymph nodes and this trafficking might result in the leaching of mycobacterial antigens from their primary site of infection to the distant sites. Thus, providing the mechanism for dissemination of mycobacterial components and hence establishing a communicating network that may have whole body manifestation. The cell integrates the lipid signalling pathways via lipid sensors that include membrane receptors and certain lipid sensing NRs such as Tr4^16^. Owing to the fact that these NRs are amenable to pharmacological modulation makes them a good therapeutic drug target to combat TB. However, we still lack an in-depth knowledge of how *M. tuberculosis* manipulates the host NRs for its survival and pathogenesis.

In this study, we monitored and determined the levels of NRs in macrophages and DCs upon *M. tuberculosis* infection to elucidate the cross-talk between the pathogen and host NRs. We chose different time points for infection to mimic the conditions where early on the host cells are either trying to eliminate the infection or later time points where the pathogen might have established a persistent infection by exploiting the host intracellular machinery. It became apparent from our findings that *M. tuberculosis* infection leads to an alteration in the levels of several members of NR superfamily in innate immune cells. Interestingly, few of the receptors (Vdr, Lxra, Pparg, Rarg, Rora, Nr4a’s, Ar, and Thrb) were commonly modulated in the studied cell types. Among these Lxra has been previously established to actively contribute towards the protective immune response against intracellular pathogen *M. tuberculosis*^17^. The epigenetic landscapes identified in *M. tuberculosis* infected human macrophages and human DCs also signifies the importance of Lxr in shaping the bactericidal response against intracellular bacteria^19,20^. In addition, 1,25-dihydroxyvitamin D that acts as a ligand for Vdr also aids in the better clearance of bacteria^25–27^. Vdr is also identified as a significant transcriptional modulator of the genes that are upregulated in human macrophages in response to *M. tuberculosis* infection^22^. Moreover, vitamin D deficiency has been correlated with increased TB susceptibility. On the contrary, Pparg increases the persistence of bacteria inside the host cells and our recent findings demonstrated that it does so by driving the alternative activation of macrophages thereby promoting the fitness of *M. tuberculosis*^12,15,29^. Furthermore, rosiglitazone, a ligand to Pparg, enhances the bacterial survival inside the macrophages^12^. In concordance with earlier studies, we found a dynamic change in the expression of Pparg upon *M. tuberculosis* infection. Interestingly, this study also identified changes in the expression of Nr4a3 and Rora, whose functions remained unknown in *M. tuberculosis* infection. While the expression of Nr4a3 was significantly decreased in macrophages and DCs, the expression of Rora was found to elevate in the infected cells. The PBMCs obtained from TB patients also showed significant changes in the expression of Nr4a3 and Rora pointing towards their plausible role in bacterial infection. Hence, it is conceivable that the dynamics of *M. tuberculosis* infection might be regulating the expression of those NRs that either enhance successful intracellular parasitisation by bacteria or are responsible for its clearance. Therefore, it will be interesting to decipher the function of other differentially regulated NRs with no reported role in *M. tuberculosis* pathogenesis.

On the other hand, we observed that few NRs are constitutively expressed at high levels without undergoing any significant changes upon *M. tuberculosis* infection but have a reported role in its pathogenesis such as Tr4. Tr4 acts as a molecular switch and by regulating the macrophage polarization towards M2 phenotype, augments the bacterial survival^12^. In a recent published study, it has been elucidated that the lipid components of mycobacterial cell wall binds to Tr4^16^. This leads to the transactivation of Tr4 thereby inducing foamy biogenesis and granuloma formation. It has now been appreciated that sometimes rather than the NR expression it is the changing repertoire of the host cellular lipids, which act as ligands for these NRs, that regulates their function. This led us to speculate that NRs with high expression might also be having a plausible role in the clearance or persistence of *M*. *tuberculosis*. However, detailed functional studies should be performed for validating their roles in *M. tuberculosis* infection. It has been revealed that Pxr, an adopted orphan NR, increases the survival of *M. tuberculosis* in its host niche (human macrophages)^23^. But in corroboration with other reports, we found insignificant expression of this NR in studied murine cell types^8,30^.

Given the fact that the current TB treatment is complex and often results in poor patient compliance, there is a compelling need for new drug regimens which are more effective with shorter treatment period. Most drugs developed till date to cure mycobacterial infections target crucial enzymatic processes in the bacterium such as transcription, translation or biosynthetic pathways like cell wall synthesis^42^. In addition to developing new novel TB-drugs that are directed against bacterial processes, there is also an increasing interest in developing therapeutics that are not directly bactericidal but target host pathways used by bacteria for either infecting, persisting or replicating inside the host^43,44^. In this connection, the findings that NRs modulate several cellular pathways that are important for bacterial survival inside the cell and their amenability to pharmacological modulation makes it interesting to explore the possibility of utilizing NRs as targets for anti-TB therapies. Adding small molecules that can regulate the activity of NRs in ways that strengthen the microbicidal properties of macrophages and DCs along with standard TB-drugs may significantly reduce the duration of TB treatment. Moreover, this adjunct therapy may also be effective in providing treatment against latent bacteria which is otherwise resistant to the anti-TB drugs.

Collectively, our study suggests the existence of a complex interplay between host NRs and *M. tuberculosis*. These NRs may provide immense opportunities as potential therapeutic targets that can regulate immune responses thus directing protective immunity against infectious diseases.

## Materials and Methods

### Human ethics statement

The project was approved by the Ethics Committee of the Government Medical College and Hospital (GMCH), Sector 32, Chandigarh, India and the Ethics and Biosafety Committee of the IMTECH, Sector 39 A, Chandigarh, India. Human PBMCs and hMDMs were obtained as described earlier^23^.

### Experimental animals

C57BL/6 mice aged 6–8 weeks were procured from the animal facility of Institute of Microbial Technology, India. Animals were housed in Biosafety Level 3 facility of the institute. All animal procedures were approved by the Institutional Animal Ethics Committee of the Institute of Microbial Technology, and were in compliance with the guidelines from the National Regulatory guideline issued by the Committee for the Purpose of Supervision of Experiments on animals (N0.55/1999/CPCSEA), Ministry of Environment and Forest, Govt. of India.

### Mycobacterial strain and culture conditions

*M*. *tuberculosis H37Rv* was cultured in 7H9 broth medium containing 5% glycerol and 0.05% Tween 80. The broth was supplemented with 10% OADC and the culture was incubated at 37 °C on a shaker. Bacterial cultures in log phase were used for infection.

### mBMDCs and mBMDMs culture

For the generation of mBMDCs, bone marrow precursors were obtained from the femur and tibia of C57BL/6 mice and treated with RBC lysis buffer. The precursors were then cultured in ultra-low attachment plates (Corning Costar) in RPMI-1640 medium (Gibco) supplemented with 10% new born calf serum (Gibco), 1% Penicillin Streptomycin (Pen Strep; Gibco), 10 ng/ml of GM-CSF (eBioscience) and 10 ng/ml of IL-4 (eBioscience), for 7 days. Non-adherent cells were harvested on day 7 and they exhibit ~90% purity as determined by the expression of CD11c. mBMDMs were derived by culturing bone marrow precursors in RPMI-1640 medium supplemented with 10% new born calf serum, 1% Penicillin Streptomycin and 50 ng/ml of GM-CSF. The non-adherent cells were replated on day 3 and kept for another 4 days. On day 7, adherent cells were harvested and they present ~98% as determined by analyzing the expression of CD11b and F4/80 by flow cytometry.

### Isolation of cells from lungs

Lungs were harvested from uninfected or *M*. *tuberculosis H37Rv* infected mice on days 7, 15, and 30. The lungs were minced and incubated with collagenase (2 mg/ml) and DNase (40 Units/ml) for 30 mins at 37 °C. After digestion, lung cells were dispersed and single cell suspension was obtained by passing it through 70 µm cell strainer. The cell suspension was washed and RBCs were lysed using RBC lysis buffer, followed by washing with 1× PBS. The cell population was enriched for CD11c positive cells using EasySep Mouse CD11c positive selection kit (STEMCELL Technologies) according to the manufacturer’s protocol.

### *M. tuberculosis H37Rv* infection of mBMDCs and mBMDMs

The mBMDCs and mBMDMs were infected with *H37Rv* at MOI of 1:5. After 4 h of infection the cells were washed to remove any unphagocytosed bacteria. The cells were then either harvested immediately after washing (considered as 0 h), 12 h, or 48 h post infection and processed for RNA isolation.

### Aerosol infection of mice with *M. tuberculosis H37Rv*

Mice were aerosol challenged (with a nebulisation system Glas-Col; Terre Haute, IN) with *M. tuberculosis H37Rv* as described previously^16^. The mice were sacrificed on day 7, 15, and 30 post *H37Rv* infection. The lungs were dissected and processed for the isolation of CD11c positive cells.

### Immunoblot analysis

Whole cell lysates were prepared and standard procedures were followed for immunoblotting. Briefly, equal amounts of total proteins were separated by SDS-PAGE on a 10% acrylamide gel and then transferred onto polyvinylidene difluoride membranes. The membranes were then blocked with 5% BSA for 1 h at room temperature, followed by overnight incubation at 4 °C with primary antibodies against Nr4a3 (AV45646, Sigma-Aldrich), Rora (sc-28612, Santa Cruz), and β-actin (sc-47778, Santa Cruz). Membranes were then washed three times with 1× PBST (pH 7.4) and incubated with appropriate horseradish peroxidase (HRP)-conjugated secondary antibodies for an hour at room temperature and developed with chemiluminescent HRP substrate.

### PCR array

The isolation of total RNA was performed from cells using an RNeasy Mini Kit (Qiagen). Using an RT^2^ First Strand Kit (Qiagen), 1 µg of total RNA was reverse transcribed into cDNA which was then subjected to RT-qPCR using RT^2^ SYBR Green ROX qPCR Mastermix (Qiagen). Customized RT^2^ Profile PCR Array (SA Bioscience) was used to determine the expression of 49 murine NRs as per manufacturer’s instructions. The QC for the RT-qPCR arrays was performed using a RT^2^ Profiler PCR Array Data Analysis version 3.5 software. The expression data which successfully passed the QC were further analysed to determine the relative fold regulation by using 2^−ΔΔCt^ method. The NRs with a Ct value greater than 32 were excluded from final analysis.

### Quantitative real-time PCR

Total RNA was obtained from cells by extraction with RNeasy Mini Kit (Qiagen) and subjected to cDNA synthesis with the Verso cDNA kit (Thermo Scientific). RT-qPCR was performed using the SYBR Green method (DyNAmoColorFlash SYBR Green qPCR Kit, Thermo Scientific). The analysis of the expressed transcripts was performed by employing 2^−ΔΔCt^ method.

### Statistical analysis

Results are represented as mean and sd. Statistical analysis was performed in Prism 7 (GraphPad). Statistical significance with multiple parameters was analyzed with one-way ANOVA followed by post-hoc Tukey’s test. The correlation analysis of NR expression between different time points has been done by using JMP11. Comparison of all pairs was performed using Tukey-Kramer HSD.

## Electronic supplementary material

Supplementary info.
