# Supplementary material for: An Accord of Nuclear Receptor Expression in M. tuberculosis Infected Macrophages and Dendritic Cells
Source: Sci Rep. 2018 Feb 2;8:2296. doi: 10.1038/s41598-018-20769-4 (PMC5797181; doi:10.1038/s41598-018-20769-4)
Supplement: Supplementary file 1 — Supplementary info. [file 41598_2018_20769_MOESM1_ESM.pdf]

Supplementary information

An Accord of Nuclear Receptor Expression in *M. tuberculosis* Infected Macrophages and Dendritic Cells.

Ankita Saini[1,2], Sahil Mahajan[1,3], Nancy Ahuja[1], Ella Bhagyaraj[1], Rashi Kalra[1], Ashok Kumar Janmeja[4], and Pawan Gupta[1,*]

[1]Institute of Microbial Technology, Council of Scientific and Industrial Research, Chandigarh-160036, India.

[2]Present address: Department of Pathology and Immunology, Washington University School of Medicine, St. Louis, MO 63110, USA.

[3]Present address: Department of Orthopedics, Washington University School of Medicine, St. Louis, MO 63110, USA.

[4]Government Medical College and Hospital, Chandigarh-160030, India.

*Corresponding author: Pawan Gupta, Institute of Microbial Technology, Council of Scientific and Industrial Research, Chandigarh-160036, India. Tel: +91-1726665221; Fax: +91-172-2690585; E-mail: pawan@imtech.res.in

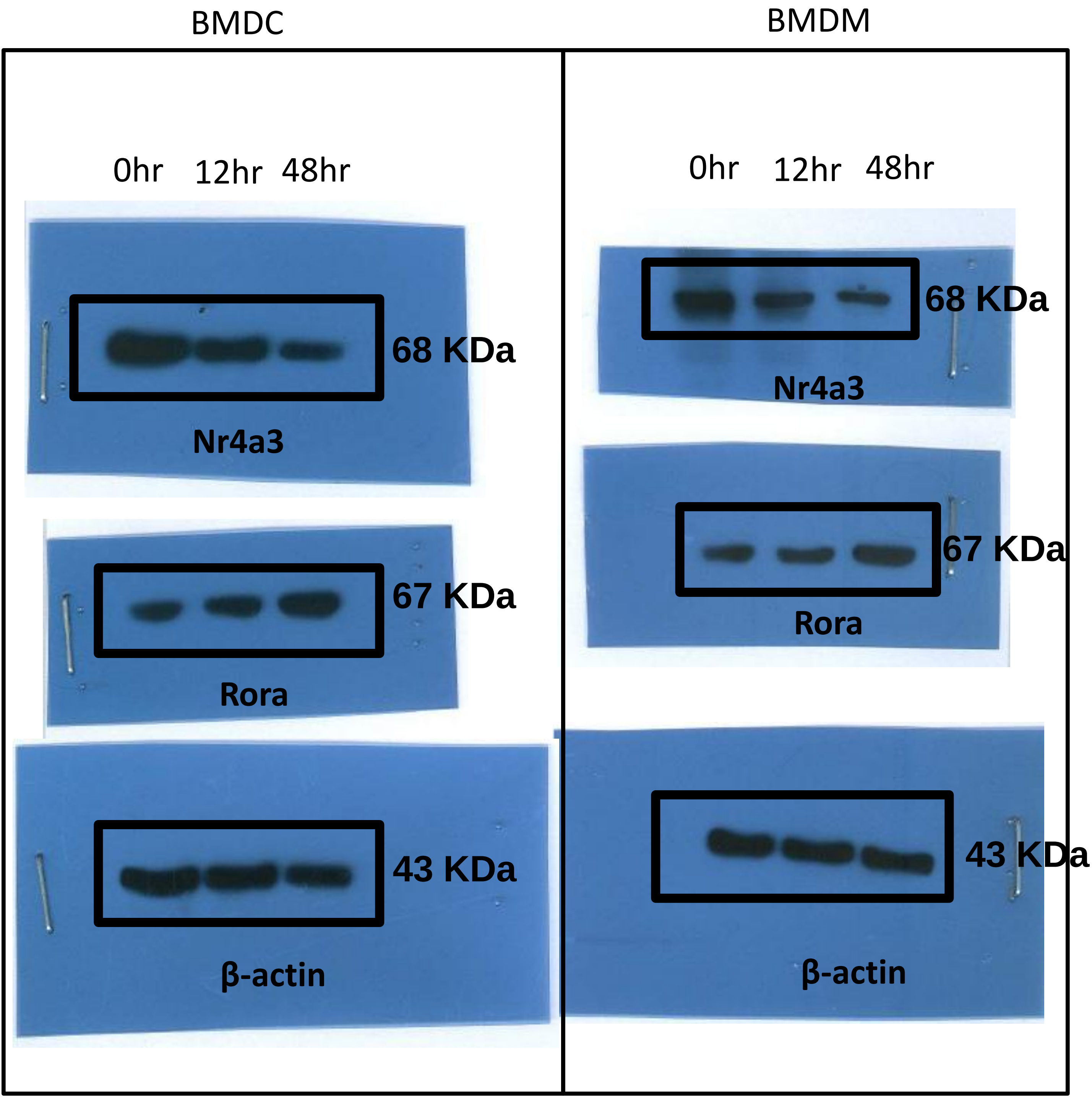

BMDC
BMDM
0hr 12hr 48hr
0hr 12hr 48hr
68 KDa
Nr4a3
68 KDa
Nr4a3
67 KDa
Rora
67 KDa
Rora
43 KDa
β-actin
43 KDa
β-actin

Figure 3L
